# Transcription Factors in the Development and Pro-Allergic Function of Mast Cells

**DOI:** 10.3389/falgy.2021.679121

**Published:** 2021-06-07

**Authors:** Mansi Srivastava, Mark H. Kaplan

**Affiliations:** ^1^Department of BioHealth Informatics, School of Informatics and Computing, Indiana University-Purdue University, Indianapolis, IN, United States; ^2^Department of Microbiology and Immunology, Indiana University School of Medicine, Indianapolis, IN, United States

**Keywords:** allergy, transcription factor, regulation, degranulation, gene expression, binding site

## Abstract

Mast cells (MCs) are innate immune cells of hematopoietic origin localized in the mucosal tissues of the body and are broadly implicated in the pathogenesis of allergic inflammation. Transcription factors have a pivotal role in the development and differentiation of mast cells in response to various microenvironmental signals encountered in the resident tissues. Understanding the regulation of mast cells by transcription factors is therefore vital for mechanistic insights into allergic diseases. In this review we summarize advances in defining the transcription factors that impact the development of mast cells throughout the body and in specific tissues, and factors that are involved in responding to the extracellular milieu. We will further describe the complex networks of transcription factors that impact mast cell physiology and expansion during allergic inflammation and functions from degranulation to cytokine secretion. As our understanding of the heterogeneity of mast cells becomes more detailed, the contribution of specific transcription factors in mast cell-dependent functions will potentially offer new pathways for therapeutic targeting.

## Introduction

Mast cells are a crucial component of the innate immune system, that originate from the pluripotent progenitor cells (Hematopoietic Stem Cells) of the bone marrow and remain in the blood before populating in the resident vascular tissues (1–3). In the tissue microenvironment, stem cell factor (also known as c-kit ligand and mast cell growth factor) and cytokines such as interleukin-3 (IL-3) direct the maturation of progenitor mast cells to perform specialized functions (4, 5). Mast cells are located throughout the body and are most abundant in the tissues that are directly in contact with the external environment such as skin, airways, conjunctiva, and mucosal tissues in the gastrointestinal tract where they regulate a wide variety of pathophysiological functions (6). Mature mast cells act as the first line of defense against pathogens and foreign proteins including allergens (3). Discovered by Paul Ehrlich in 1879, mast cells are recognized for their versatile role in a range of immunological responses in health and disease (7). Mast cells are rich in secretory granules containing prostaglandins and histamine, and this distinguishes them from other immune cell types (8). Differentiation and phenotypic characterization of mast cells is dependent on several factors including environmental stimuli, cytokines, and transcription factors (9) that control gene expression and influence the phenotypic and functional identity of cells.

Mast cells respond to a multitude of extracellular signals through activation of receptors that regulate their functions and survival in tissue microenvironments (10, 11). First described in 1970, high affinity IgE crosslinking is the most widely understood mechanism of mast cell activation during allergic response and anaphylaxis (12–15). Studies have demonstrated three subunits in FcεRI receptor: α subunit that binds to IgE, β subunit, and a dimer of disulfide-linked γ subunits (16). Activation of mast cells occurs after monomeric IgE bound to FcεRI via the Fc region (C_H_3 domain) interacts with the antigen through the Fab region, crosslinking FcεRI subunits and initiating downstream signal transduction (17, 18). The downstream signaling cascade results in biphasic responses from mast cells (19, 20). In the first phase there is rapid degranulation and release of mediators such as histamine, while in the second phase there is release of inflammatory mediators such as cytokines, chemokines, prostaglandins and leukotrienes (21) and proteases such as chymase and tryptase from cytoplasmic granules (10, 11). Recent studies have shown that suppressing IgE- FcεRI crosslinking by blocking translation of FcεRI β-subunit or modulating splicing events and inducing exon skipping could be a potential therapeutic strategy as tested in a mouse model of allergic dermatitis (22).

In addition to FcεRI, mast cells also express Toll-like receptors (TLRs) that interact with pattern-associated molecular patterns (PAMPs) and have a notable contribution in mast cell activation. There are 10 TLR family receptors that include both cell surface receptors (TLR 1–2, 4–6) and endosomal receptors (TLR3, 7–9) (23). Previous studies demonstrated that mast cells respond to TLR ligands such as lipopolysaccharide (LPS) and peptidoglycan (PGN) that in turn can provoke mast cell degranulation via TLR2 and TLR4 after IL12 induction via activation of PI3K/Akt signaling cascade (23, 24). It has also been shown that TLR ligands can cause synergistic activation of mast cells via FcεRI, thus augmenting the response to antigenic stimulation (23). Response to individual TLR ligands differentially activates mast cells and results in secretion of distinct cytokines. For example, TLR2 activation results in the production of IL-4, IL-5, IL-6, and TNFa, while TLR4 activation induces secretion of IL-1b, IL-6, IL-13, and TNFa (23, 25). Interestingly, IgE induced mast cell response via FcεRI crosslinking was reduced following pre-exposure to dual TLR2/7 ligands; however, simultaneous exposure to both TLR ligands and FcεRI stimulation enhanced the cytokine secretion from mast cells (26–28).

A vast array of chemokines and cytokines have also been found to have profound impact on mast cell activation and response to stimuli. Growing evidence shows that mast cells express a range of chemokine receptors including CCR1, CCR3-5, CXCR1-4, and CX3CR1 (29). Response to chemokines by these receptors induces mast cell migration to tissue microenvironment for maturation and allergic functions (30). Cytokines such as IL-33 have been shown to provoke mast cell activation leading to degranulation and secretion of chemokines and cytokines that contribute to allergic responses (31, 32). Previous studies report that IL-33 mediates bronchial constriction in mice through secretion of serotonin from mast cells, thus indicating a possible mechanism of mast cell activation (33). The response to physiological stimulus and the effector function of mast cells requires the activity of several transcription factors such as GATA1 & 2, MITF, PU.1, STAT5, and BATF (34, 35). TFs like AP-1 are immediately induced following IgE-Ag stimulation leading to degranulation from cytoplasmic secretory granules (36). Other critical transcription factors like Ets homologous factor (Ehf) and Interferon regulatory factor-8 (IRF8) are associated with mast cell development and functional identity. These transcription factors regulate mast cell genes crucial for development of allergic responses.

In this review we discuss recent insights on the transcription factors that play a crucial role in the development as well as differentiation of mast cells localized across tissues. We also shed light on the intricate network of transcription factors that impact the functions of mast cells during allergic inflammation. Since transcription factors play several regulatory roles in the physiological function of mast cells, they constitute major targets for therapeutic applications. Thus, a thorough understanding of the transcriptional regulation of mast cells will pave potential directions for clinical targeting in mast cell mediated allergic diseases.

## Transcription Factors Regulating Mast Cell Development

Transcription factors play critical roles both in the development of mast cells and the regulation of genes within mature mast cells. Table 1 summarizes the key transcription factors and the impact of their deficiency on mast cell phenotypes. In the following sections we focus on specific transcription factor families and detail how they contribute to mast cell development.

**Table 1:** Mast cell phenotypes in transcription factor mutant models.

**Transcription factor**	**Mutant model**	**Effect on mast cell phenotype**	**Reference**
GATA1	GATA1^low^ mutant mice (lacking the first enhancer (DNA hypersensitive site I) and the distal promoter)	Morphological abnormality in mast cells from peritoneal lavage and connective tissue	(37)
GATA2	BMMCs with deletion of the GATA2 DNA binding domain from GATA2^flox/flox^ mice (GATA2ΔCF)	Loss of mast cell identity marked by downregulation of mast cell-specific genes (c-kit) and upregulation of myeloid genes	(38)
Ehf	BMMCs transfected with retroviral vector encoding FLAG-tagged mouse Ehf Ets homologous factor (Ehf)	Significant suppression of FcεRI and c-Kit expression induced by TGF-β1 in mast cells	(39)
PU.1	siRNA mediated knock down of PU.1 in BMMCs	Diminished IgE-mediated activation of mast cells, and significant reduction of the the Syk and FcεRIβ mRNA levels	(40)
MITF	MITF^wh/wh^ mice with two copies of the MITF gene with a single amino acid mutation at the basic domain (DNA binding domain)	MITF mutant mast cells switched to “basophil-like” cells and lost c-Kit and IL-4 receptor α chain expression	(41)
BATF	BATF germline knock out mice	Defect in OVA-specific IgE and IL-3 levels and mast cell development	(42)
STAT5	Stat5 knock out BMMC with IgE plus antigen stimulation	Mast cells exhibited significant reduction in IgE-mediated degranulation and cytokine secretion, due to decreased cytokine mRNA stability.	(43)
IRF8	IRF8 knockout mice	Loss of mast cell progenitors and inability to efficiently differentiate into mast cells	(44)
ATF3	ATF3 knockout mice	diminished proliferation and maturation with enhanced apoptosis of mast cells	(45)
STAT6	STAT6 knockout mice	No effect on IL-4 production in mast cells	(46)

### GATA Family

The GATA transcription factors are a family of zinc finger proteins that are named for their recognition and binding of the consensus DNA sequence (T/A)GATA(A/G) (47, 48). GATA TFs have a common zinc finger DNA binding domain which is required for recognition and binding to the consensus sequence and that stabilizes the complex by interaction with other proteins (49–51). However, these transcription factors show variation in their N and C terminal regions that are responsible for transcriptional activation (52, 53). The GATA factors have the ability to bind both DNA and proteins and form a transcriptional complex by recruiting chromatic remodeling proteins to facilitate the transcriptional regulation of their target gene (54, 55). Based on their sequence homology, GATA proteins are divided into two major subfamilies, GATA 1–3 expressed in hematopoietic stem cells and GATA 4–6 expressed in mesoderm and endoderm derived tissues (35, 48, 56).

Mast cells express GATA-1 and GATA-2, and both transcription factors are required for mast cell differentiation and development (57). GATA1^low^ mutant mice (that lacks the first DNase hypersensitivity site/enhancer and the distal promoter of the GATA-1 gene) exhibited morphologically abnormal mast cells in peritoneal lavage and connective tissue indicating abnormal mast cell development (37). Importantly, siRNA mediated knockdown of GATA1 did not affect GATA2 expression in cultured mast cell line (P815 cells) and BMMCs, indicating a lack of cross-regulation between both transcription factors (58). However, ChIP assays revealed that both GATA factors bound to conserved GATA sites in BMMCs (58). Interestingly, studies on BMMCs from mice lacking the GATA2 DNA binding domain (GATA2ΔCF), showed loss of mast cell identity marked by downregulation of mast cell-enriched genes, including c-kit. This study also found that GATA2-deficient BMMCs exhibited characteristics of immature myeloid-like cells. Thus, GATA2 exerts a fundamental regulatory role in differentiated mast cells (38).

GATA2 also acts more broadly in mast cell gene regulation and a recent study illustrated that GATA2 regulated many mast cell genes by promoting chromatin accessibility at super enhancer region of these genes, thereby maintaining cellular identity (59). A study on human airway mast cells from patients with asthma and chronic rhinosinusitis with nasal polyposis (CRSwNP) provides evidence for distinct inflammation driven transcription factor phenotypes using single cell RNA-sequencing (60). The study identified GATA2 as a highly enriched transcript in the nasal polyp MCs. A recent study has shed light on the role of GATA2 in the regulation of E-cadherin expression in mast cell and basophils using publicly available GATA2 ChIP-sequencing data. The study recognized a highly enriched site of GATA2 in the promoter of E-cadherin in BMMCs, indicating a potential regulatory role of GATA2 on E-cadherin mediated mast cell differentiation (61). Notably, GATA-2–mediated E-cadherin expression is recognized as a signature for early progenitor cells that are primed to become mast cell and basophil lineages during hematopoiesis.

Contrary to the function of GATA1 and GATA2 the GATA3 transcription factor suppresses the activation of mast cells in an airway rhinitis mouse model (62). This study also found that microRNA-135a (miR-135a) binds to GATA3 and higher expression of this miRNA negatively regulates mRNA and protein levels of GATA3. However, a mechanistic understanding of miR-135a through GATA3 is required to identify its therapeutic potential in allergic rhinitis.

### Ets Family: Ehf and PU.1

Ets (E26) proteins are a family of transcription factors that are named after v-ets oncogene originally found in avian retrovirus (63). Ets factors are characterized by the presence of conserved DNA-binding domain, the Ets-domain that specifically recognizes sequences that have “GGA” core trinucleotide (64). The Ets domain also facilitates interaction with other co-factors such as CBP/p300 histone acetyltransferases and the Sp1 transcription factor that cooperatively regulate functions of Ets proteins (65–68). Around 30 Ets family proteins have been identified ranging from flies to humans (69, 70).

Previous studies showed that TGF-β/Smad signaling in mouse BMMCs upregulated Ets homologous factor (Ehf) expression (39). The study also demonstrated that overexpression of Ehf in BMMCs caused transcriptional repression of mast cell genes such as FcεRIα, FcεRIβ, and c-Kit leading to suppressed degranulation and cytokine secretion from these cells. Authors further provided evidence that stable expression of Ehf in BMMCs reduced mRNA levels of key transcription factor such as GATA1, GATA2, and STAT5B suggesting an intricate network of TFs regulating mast cell functions. The findings further indicate that decreased expression of STAT5B may be an important contributing factor to the suppression of cytokine production by BMMCs.

PU.1, another member of the Ets transcription factor family, is essential for the development of mast cells (35, 71, 72). PU.1 is indispensable for mast cell homeostasis and differentiation evident by failure of PU.1 knock out fetal liver cells to differentiate into mast cells in the presence of SCF and IL3 (73). In a study involving PU.1 siRNA knock down in BMMCs, a significant reduction in the IgE mediated mast cell activation was accompanied with suppression of Syk and FcεRIβ mRNA levels (40). Studies involving siRNA mediated downregulation PU.1, GATA1, and GATA2 in the human mast cell line LAD2 showed a significant reduction in the expression of FcεRI which was further supported by suppressed degranulation from LAD2 cells (74). ChIP assays from the same study illustrated an enriched binding of all three transcription factors on the promoter region of FcεRI, suggesting a crucial role of these TFs in mast cell activation. These findings strongly indicate an indispensable role of these TFs in the transcriptional regulation and promoter binding of FcεRI gene.

### MITF

MITF (Microphthalmia transcription factor) is a helix-loop-helix (HLH) domain containing factor with a basic and leucine zipper domain essential for mast cell development as evident by severely reduced mast cell numbers in MITF mutant mice (75–77). High expression of MITF is crucial for differentiation of common basophil/mast cell committed progenitors (BMCPs) into mast cells while a loss of MITF leads to basophil lineage (78). Recently, RNA-sequencing from LPS stimulated BMMCs has also demonstrated high expression of MITF transcription factor (79). Multiple MITF isoforms have been identified in various cell types, however mast cells predominantly expressed MITF-a, MITF-e, and MITF-mc (80, 81). Presence of multiple isoforms in mast cells have been linked to diverse biological functions including restoring granular morphology, mast cell differentiation and migration (81).

A recent review summarized the positive and negative regulators of MITF in mast cells and their impact on mast cell biology (82). MITF is a pleiotropic transcription factor as MITF-mutant mice displayed a number of phenotypic defects such as retinal degeneration, hearing loss, osteopetrosis and abnormal pigmentation (82). Parallel to defects in mast cell development, MITF-deficient mice also have defects in osteoclasts and melanocytes (75). Studies examining MITF-interacting proteins identified protein kinase C interacting (PKCI) protein 1 and protein inhibitor of activated STAT3 (PIAS3) as inhibitors of MITF activity in mast cells (75, 83, 84). A recent study on human cord blood–derived MCs (CBMC), evaluated IL-4 regulation of the polyp mast cell transcriptome. This study found that IL-4 stimulation downregulated MITF transcript levels, suggesting that IL-4 might be a critical cytokine that exerts transcriptional regulation on MITF in the cord blood derived MCs (60).

MITF regulates several mast cell genes that play an important role during differentiation and cell activation. Some of the key genes include granzyme B (GrB) that acts as the key cytotoxic mediator and tryptophan hydroxylase (TPH), the rate limiting enzyme that catalyzes tryptophan to serotonin, required for mast cell mediated immune response (85, 86). MITF also regulates expression of several mast cell proteases (mMCP-2,−4,−6, and−9), cathepsin G and c-kit through a locus control region (82, 87). Studies on transformed mast cells show that c-kit signaling upregulates MITF protein expression without affecting its mRNA levels (88). Two miRNAs including miR-539 and miR-381 were found to repress MITF expression in a mastocytosis cell line. Further, studies in this direction are required to identify potential MITF regulators that could be used as therapeutic targets to modulate mast cell functions.

### BATF

BATF (basic leucine zipper transcription factor, ATF-like), belongs to the AP-1 family of transcription factors and is shown to be predominantly expressed in cells of hematopoietic origin (89). BATF is characterized by a basic leucine zipper and regulates differentiation and function in several lymphocyte lineages including class switch recombination in B cells (90–92). BATF also contributes to the Th2- and Th9-dependent responses in mouse models of asthma (42, 89). In the context of mast cell function, BATF knockout mice have a deficit in OVA-specific IgE levels, IL-3 secretion, and mast cell development (42). BATF knockout mice sensitized with Ova had significantly reduced numbers of lung mast cells expressing the IL-3 receptor α-chain (93). Similar observations were found in BMMCs, suggesting that BATF plays an important role in mast cell development in the lungs. Recently, an IL-4-BATF axis was identified in the regulation of IL-9 producing mucosal mast cell (MMC9) function during IgE mediated food allergic reactions (94). Using RNA-seq analysis, the study identified 410 gene transcripts that were regulated by IL-4 signaling, including IL-9 and BATF in MMC9. The results suggest a key role of BATF in modulating the transcriptional program in mucosal mast cells.

### STAT5

Signal transducer and activators of transcription (STAT) are a seven-member family of proteins that are evolutionarily conserved and regulate gene expression downstream of activated cytokine and hormone receptors (95–97). STAT proteins are widely recognized as critical mediators of the JAK-STAT pathway that regulate gene expression involving complex interaction with several transcriptional activators, repressors and chromatin modifying proteins following their nuclear phosphorylation (98). Among all members, two closely related factors STAT5A and STAT5B are of particular interest because of their functional involvement in various cellular processes including but not limited to cell differentiation, survival, proliferation and oncogenesis in cell type specific manner (99). STAT5A and STAT5B share 96% amino acid sequence similarity (100). However, differences in both factors are reported in their extreme 5′ and the 3′ exons, specifically in STAT5B two additional 5′ exons that code for alternative promoters have been identified (100). Several studies have shown that STAT5 proteins are critical regulators of mast cell development, function and survival (43, 101, 102). Both factors have been recognized as critical factors that regulate mast cell development and survival (103). In mast cells, IL-3 and c-kit receptor stimulation activated STAT5 (104). In addition to these receptors, IgE-crosslinking induces rapid activation of STAT5 protein that in turn regulated mast cell functions (43). These findings were further confirmed with STAT5-deficient (STAT5KO) MC, that demonstrate a reduction in IgE-mediated degranulation, and subsequent cytokine secretion and leukotriene production compared to wild type cells. Notably, it was observed that STAT5KO MC induced normal levels of cytokine mRNA following IgE crosslinking, however, a rapid degradation of these mRNA was observed over time, indicating that STAT5 is a critical factor in mRNA stability (43).

The same research group also demonstrated that STAT5B knockout cells exhibited decreased sensitivity to IgE mediated systemic anaphylaxis response marked by decreased production of IL-6 and IL-13 compared to wild type cells (105). The study also found that STAT5B phosphorylation on serine residues occurs via a Src-independent pathway that requires PI3-kinase function. These results provide insights into the role of STAT5B factor during mast cell mediated inflammatory response. Therefore, further understanding the mechanism of STAT5B phosphorylation could facilitate development of new targets for combating mast cell disorders.

STAT5 tyrosine phosphorylation was shown to be rapidly and transiently induced by activation of the high affinity IgE receptor, FcεRI on mast cells (106). Using antigen-stimulated mast cells, the study showed that STAT5 co-localizes with FcεRI accompanied by depletion of cholesterol from the cell membrane, that in turn reduced STAT5 tyrosine phosphorylation. More mechanistic insights into STAT5 mediated mast cell functions were provided from this study using pharmacological inhibitors and knock out models. It was shown that Fyn kinase induced IgE-mediated STAT5 activation independent of other kinases including PI3K, Akt, Bruton's tyrosine kinase, Syk, and JAK2 (102, 107). Together these studies illustrate distinct role of STAT5A and STAT5B in mast cell mediated inflammatory functions.

### AP-1

AP-1 (activating protein-1) are dimeric DNA binding complexes of Fos, Jun or ATF2 subunits that dimerize through leucine zippers and bind to AP-1 binding site defined as TGA(C/G)TCA (34, 108–111). As discussed above, BATF also forms complexes with AP-1 family members. Studies on AP-1 gene knockout mice suggest that members of AP-1 family may regulate distinct genes and therefore exert a variety of biological outcomes (112–114). Members of AP-1 family have been widely recognized for their role in cell proliferation, survival and apoptotic functions (111, 115–117).

Previous studies demonstrated that mast cell IL-3 but not IL-4 induced DNA binding activity of AP-1, indicating differential involvement of AP-1 in the cytokine mediated response of mast cells (118). Stem cell factor (SCF), a critical regulator of mast cell growth and functions has been shown to induce AP-1-dependent production of IL-6 via interaction with IL-6 promoter via MAPK kinase 3 activity (119). Earlier reports on IgE-Ag stimulated murine fetal-liver-derived mast cells showed that protein kinase C (PKC) enzymatic activity enhanced the accumulation of c-Fos mRNA and protein but only the protein of c-Jun, defining a PKC dependent regulation of AP-1 activity in mast cells (120). In human intestinal mast cells there is rapid induction of c-Fos and c-Jun components of AP-1 following FcεRI crosslinking (121). Activation of AP-1 regulates the expression of cytokine genes which further amplifies mast cell response to IgE receptor activation.

### Additional Transcription Factors That Regulate Mast Cell Functions

In addition to the transcription factors described above, several other factors have been implicated in mast cell development and function. Studies have shown that interferon regulatory factor-8 (IRF8), a transcription factor crucial for development of myeloid cells also impacts the activity of mast cells (44, 122). IRF8 knock out mice have a severe loss of mast cell progenitors and *Irf8*^−/−^ granulocyte progenitors failed to efficiently differentiate into mast cells (44). The study also found that GATA2 which is also essential for mast cell differentiation, is a downstream target of IRF8.

A recent study identified enhancers regulating the expression of IL-13 gene in response to IgE receptor crosslinking in mast cells (123). This study identified potential enhancers on mouse IL-13 gene using histone modification marks (H3K4me3) ChIP-seq. Interestingly, a cluster of transcription factors including the NFATC2, STAT5, GATA2, AP1, and RUNX1 were found to have binding sites at the proximal *Il13* enhancer. Another cluster consisting of EGR2 binding sites was identified at the distal *Il13* enhancer. Binding of these transcription factors to the *Il13* gene locus played a prominent role in responding to signals triggered by antigenic stimulation. Mutations in the individual TF binding site revealed that GATA2, AP1, and RUNX1 binding sites were critical for mediating the response to IgE crosslinking.

RNA-sequencing of peritoneal mast cells in a recent study demonstrated high expression of several transcription factors that have not yet been associated to mast cell development. These factors included Runx1, Runx3, stress-induced transcription factor CREB3l1, TOX2 (TOX High Mobility Group Box Family Member 2), Crtc3 (CREB-regulated transcription activator 3), Atf7ip (activating transcription factor 7-interacting protein 1 and Tal1 (basic helix-loop-helix transcription factor. Another study that examined the factors required for induction of IL-9 producing MMC9 cells in a food allergy model illustrated the essential requirement for STAT6 in the development of these cells, although STAT6 is not required for mast cell development in general (124, 125). A study using STAT6 knock out mice revealed that STAT6 is not essential for mast cell IL-4 production (46). Furthermore, a mast cell specific isoform of STAT6 has been described that acts as transcriptional repressor of IL-4, which could act as a negative feedback mechanism to provide protection from IL-4 mediated inflammation in mast cells. Further studies are required to establish the role of these transcription factors in mast cell functions and allergic response.

## Transcription Factor Network in Mast Cells

As suggested from our summary of a number of the factors above, there is interplay among the transcription factors in the form of hierarchical expression and cooperativity in regulation of specific genes. Mast cell development has been linked to several transcription factors and interplay between these factors has been documented in several studies (34, 35). PU.1 and GATA2 have exhibited a cooperativity in regulating transcription of mMC-CPA (mouse mast cell- Carboxypeptidase A) gene, an early marker of mast cell lineage cells. Similar combinatorial regulation was observed by both factors on the IL-4 gene enhancer, indicating that cooperative regulation by these transcription factors is essential for promoting several mast cell functions (126). It is worth noting that, unlike the antagonistic effect of PU.1 and GATA2 seen during erythroid and monocyte development, these factors show cooperativity for mast cell lineage development (127, 128). ChIP analysis revealed a shared region between GATA2 and PU.1 at a +10.4 kbp region downstream of Ms4a2 gene locus that encodes for β chain of the high-affinity IgE receptor (FcεRI), while a −60 bp region exclusively occupied by GATA2. The study also elucidated that ablation of PU.1 interfered with the binding of GATA2 to both the regions (129). Thus, the coregulation of these TFs plays a central role in the modulating the expression of mast cell genes by binding to common regulatory regions.

A recent study examined the role of GATA1 and GATA2 in the regulation of mucosal mast cell specific protease genes *Mcpt1* and *Mcpt2* and demonstrated that suppression of GATA1 and GATA2 significantly reduced the mRNA levels of protease genes in BMMCs (130). The study also elaborated that TGF-b stimulation upregulated *Mcpt1* and *Mcpt2* genes in BMMCs, while suppression of the transcription factors SMAD2 and SMAD4 by siRNA markedly reduced the expression of both genes. To define this effect, the authors further examined the association between GATA2 and SMAD providing evidence that acetylation of histone H4 of the conserved GATA-SMAD motif localized on the *Mcpt1* and *Mcpt2* genes and GATA2 recruitment were increased by TGF-b stimulation. Importantly reporter assays from the study demonstrated GATA-SMAD motif dependent upregulation of GATA2 transactivation. GATA2 expression is shown to be regulated by other transcription factors such as IRF8 to induce development of mast cells and basophils (44, 122). Interestingly, a STAT5-GATA2 axis was previously demonstrated in pre-BMPs (Basophil mast cell progenitors), where STAT5 directly targeted and induced the expression of GATA2. This cooperativity between STAT5 and GATA2 further induced two downstream transcription factors, C/EBPα which is critical for development of basophil lineage and MITF, which is essential for mast cell lineage (131). Another study used genome wide gene expression profiling to provide evidence for antagonistic regulation between C/EBPα and MITF transcription factors, revealing that C/EBPα represses mast cell development by directly suppressing MITF transcription (41).

**Figure 1:**
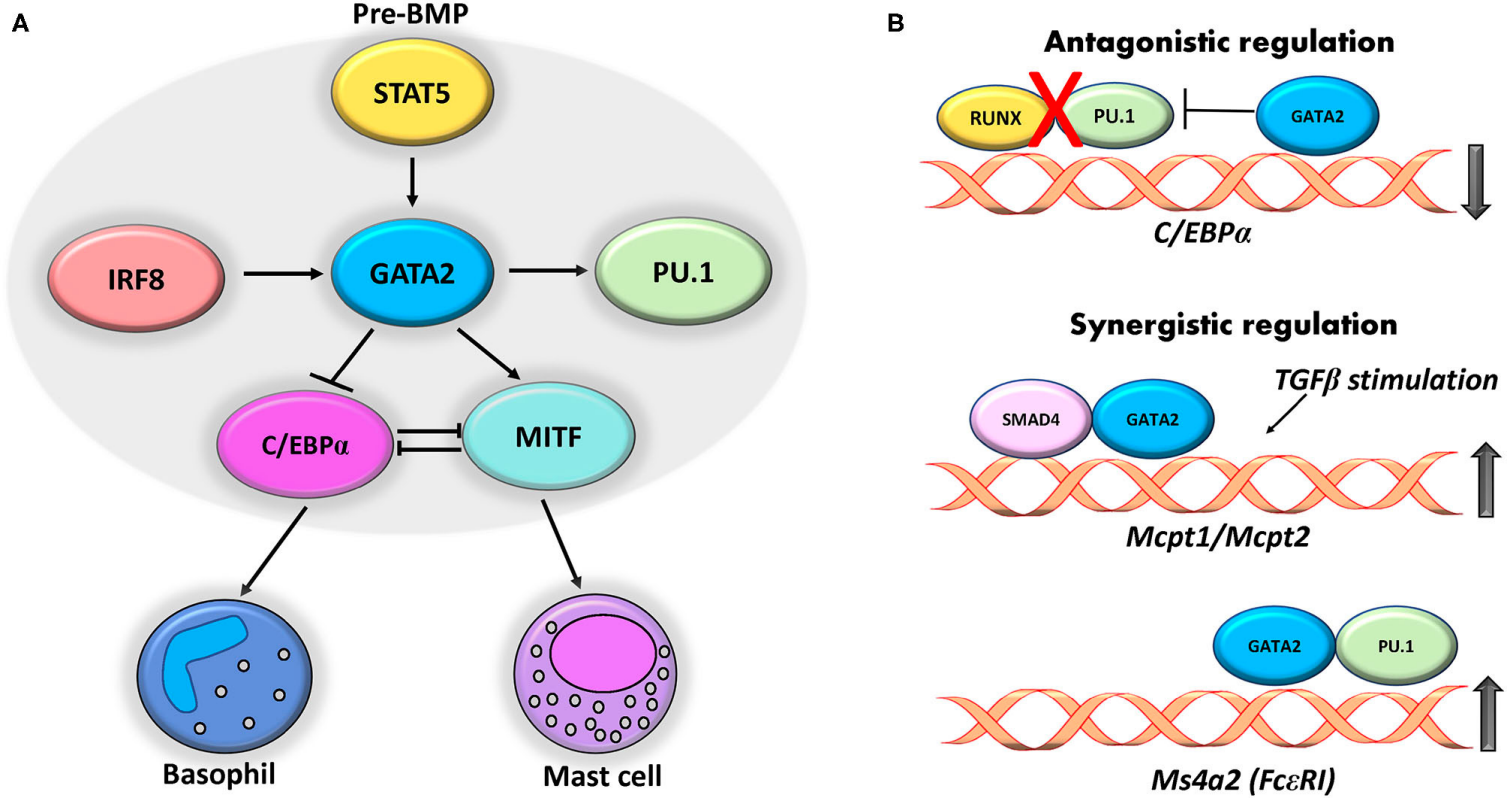
Cross regulatory interplay between transcription factors. **(A)** GATA2 expression in pre-BMPs is induced by STAT5 that in turn regulates expression of C/EBP and MITF that give rise to basophil and mast cells. IRF8 and PU.1 act synergistically with GATA2 to regulate the expression of mast cell genes. **(B)** GATA2 binds to +37 kb region on C/EBPα gene and ablates binding of RUNX1 and PU.1 thereby repressing its expression. Cooperative regulation of GATA2 with SMAD4 and PU.1 induces expression of mast cell protease (Mcpt1 and 2) and Ms4a2 respectively in mast cells.

The underlying mechanism of this transcription factor cooperation has been described in previous reviews (34, 35). Studies show transcriptional hierarchy in which PU.1 and GATA2 synergistically bind to the GATA1 gene regulatory element to activate its expression in mast cells (71, 126). Cooperative regulation between PU.1 and GATA2 on physically distinct regulatory regions of the IL-4 enhancer has also been shown previously (132). Cooperative regulation by GATA2 was observed in a study that used BMMCs lacking DNA binding domain of GATA2 (GATA2ΔCF) (38). ChIP assay from this study revealed that GATA2 directly targeted the +37 kb region of the C/EBPα gene and impedes the binding of RUNX1 and PU.1 to the neighboring region, thus modulating mast cell response. These cross regulatory networks of transcription factors play a prominent role in defining the mast cell lineage (Figure 1A). The cross talk between these factors regulates several key genes such as *Mcpt1, Ms4a2*, and *Cebpa* (Figure 1B).

AP-1 proteins crosstalk with other critical transcription factors in mast cells to exert a synergistic response during allergy and inflammation. NFAT and AP1 transcription factors synergistically activate TNFα transcription in mast cells following IgE plus antigen stimulation independent of phosphoinositol-3-kinase signaling (133). Another group demonstrated that IL-33 stimulation of mast cells synergistically activates AP-1 and NFAT that together enhance cytokine production from stimulated cells (134). Masuda et al. demonstrated direct interaction of AP-1 with GATA-1 and GATA-2 proteins that results in enhanced binding of AP-1 to IL-13 promoter region causing a surge in IL-13 production in mast cells (135). ATF3, a member of ATF/CREB (cyclic AMP response element-binding) family transcription factors, binds to the components of AP-1 family and its activity differs with the binding partners (136). For instance, heterodimerization of ATF3 and c-jun activates their target genes but a separate set of genes is activated by dimerizing with JunB (137). ATF3 has been described as an important regulator of mast cell functions. BMMC derived from ATF3-deficient mice exhibited a lack of response to IL-3–induced maturation signals, resulting in diminished proliferation marked with enhanced apoptosis and impaired activation of the Akt kinase (45). These studies suggest that a network of AP-1 with other vital transcription factors intricately regulates activation and pro-allergic response of mast cells. Further dissecting the transcriptional network and mast cell genes targeted by these transcription factors would provide an important resource toward development of targeted gene therapies for mast cell driven allergic diseases.

## Regulation of Mast Cell Genes

### Proteases

Proteases secreted from mast cells, enhance tissue permeability to enable infiltration of other immune cells to amplify allergic responses (138). Notably, 30–50% of the total secretory protein in mast cells is constituted by proteases (139). In humans, activation of mast cells significantly increases the secretion of β-tryptase during degranulation (140). Because of their involvement in allergic and inflammatory disorders, regulation of mast cell tryptases by transcription factors have been extensively studied (52). Previous reports suggest that transcription of tryptase genes is regulated by MITF transcription factor (141). The study used mutant constructs of tryptase promoter to show that two E-box (CANNTG) motifs between −817 to −715 and −421 to −202 on tryptase locus contribute to the transactivation of tryptase gene via MITF transcription factor (141).

**Figure 2:**
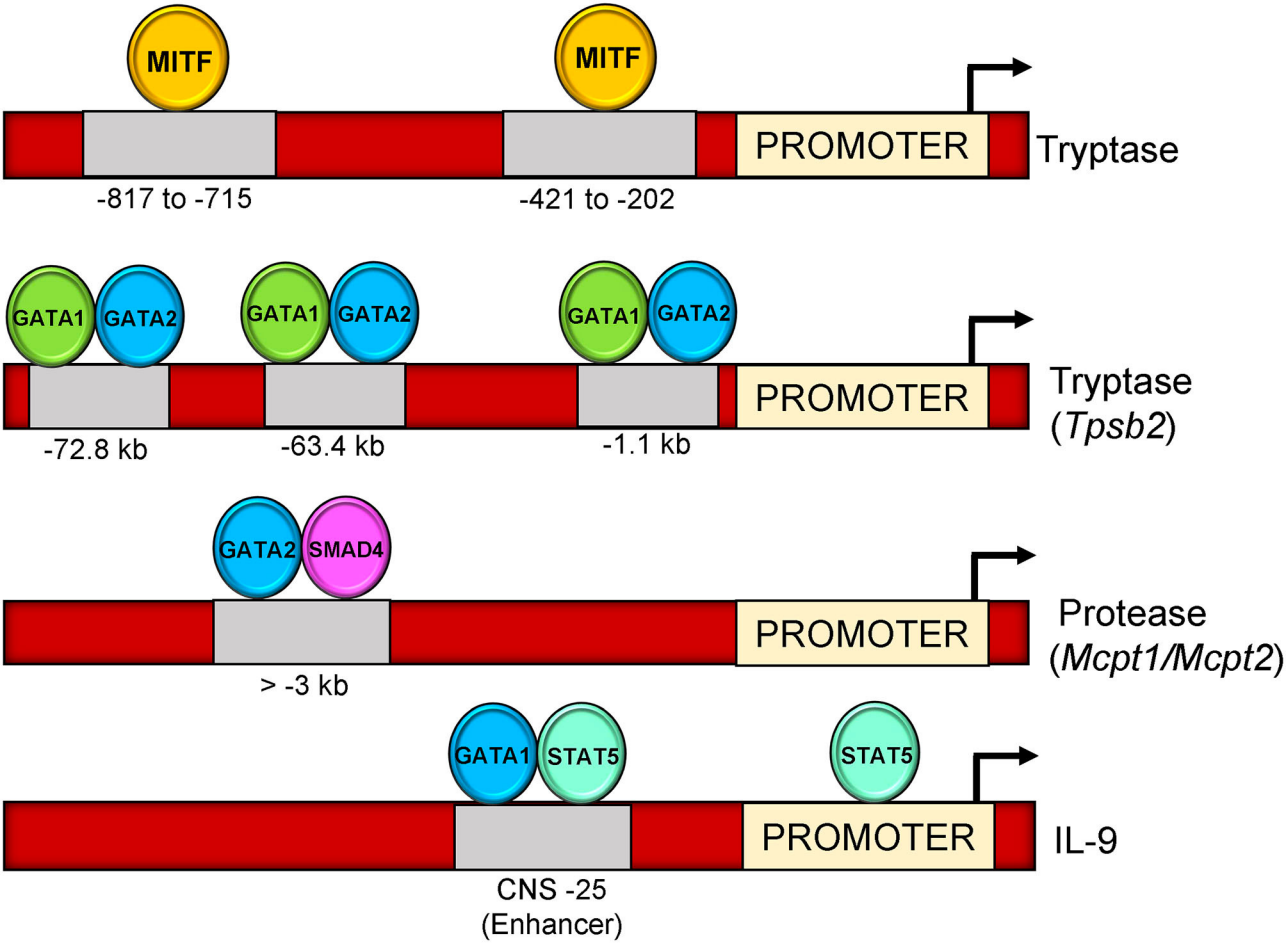
Binding sites of transcription factors on mast cell genes (Tryptase, Tpsb2, Mcpt1, Mcpt2, and IL-9). MITF binds on two locations upstream of tryptase gene promoter (−817–715 and −421–202) on E-box motif (CACCTG). GATA1 and GATA2 bind three locations upstream of Tpsb2 gene (−72.8, −63.4, and,1.1 kb). GATA2-SMAD4 occupy a region at >-3 kb upstream of Mcpt1/Mcpt2 promoter. GATA1-STAT5 bind to a CNS-25 regulatory region at IL-9 gene loci.

GATA factors also contribute to mast cell-specific tryptase gene regulation. A recent study showed that siRNA Targeting of either GATA1 or GATA2 into bone marrow derived mast cells contributes to a significant loss of mast cell tryptase gene expression (*Tpsb2* and *Tpsg1*) (142). ChIP assay from the same study revealed a 500 kb region in the 5′ end of the tryptase loci referred to as “region A” that contains binding sites for both GATA1 and GATA2 (−72.8, −63.4, and −1.1 kb regions) (Figure 2). A recent study investigating the role of GATA1 and GATA2 in regulation of tryptase gene expression in BMMCs hypothesized that the coordinated activity of both GATA1 and GATA2 could contribute to synergistic regulation on the tryptase gene locus (142). The study emphasized that GATA1 and not GATA2 plays a prominent role in tryptase gene regulation, and that GATA1 could have a role in GATA2 mediated activation of the tryptase gene locus at a −72.8 kb region.

Transcriptional regulation of the mucosal mast cell proteases *Mcpt1 and Mcpt2* genes after TGF-β stimulation was examined recently. Authors found a suppression of both *Mcpt1* and *Mcpt2* gene expression upon siRNA targeting SMAD2 or SMAD4 transcription factors, while a moderate reduction in presence of SMAD3 siRNA in BMMCs (130). Similar to SMAD transcription factors, knockdown of GATA proteins (GATA1 and GATA2) also diminished the expression of *Mcpt1* and *Mcpt2* in BMMCs in the same study. Specifically, the distal regions of both genes harbored the conserved GATA-SMAD motifs and binding of TFs to these regions was enhanced by TGF-β stimulation (130).

### Cytokines

Stimulation of mast cells via their high affinity IgE receptor (FcεRI), results in the release of inflammatory mediators and cytokines including IL-4, IL-5, IL-13, and TNF-α that mediate a variety of allergic functions (143, 144). Due to their critical role in modulating mast cell functions, the transcriptional network that controls mast cell cytokine genes has been extensively studied.

Interleukin 4 (IL-4) was the first secreted cytokine identified in mast cells (145). Several studies have shown that induction of high affinity IgE receptor (FcεRI) stimulated IL-4 secretion, which then augmented the capacity of mast cells to secrete other cytokines such as IL-5 and IL-13 (146–148). IL-33 and lectins also stimulated IL-4 secretion from mast cells (31, 149, 150). Importantly, it was shown that mast cells produce IL-4 independent of STAT6 (124). Furthermore, a study describes an isoform of STAT6 expressed in mast cells that represses IL-4 transcription. STAT6-mediated repression is anticipated to protect tissues from IL-4 mediated inflammation caused by mast cell stimulation during an infection (46).

It is worth noting that, distinct from IL-4 regulation in T cells, several transcription factors such as GATA1/2, NFAT2, PU.1, and Ikaros (zinc finger transcription factor) have been identified to bind at the gene locus and induce IL-4 production by mast cells (132, 151, 152). Similar to IL-4 gene regulation, several studies have examined transcription factors that regulate IL-13 transcription. One of the studies identified the role of NFAT1 as the major transcriptional regulator of IL-13 expression in mast cells (153). Although, NFAT2 is also expressed in mast cells, it is less likely to exert transcriptional control on the IL-13 gene than NFAT1. Importantly, it was found that the differential regulation between NFAT1 and NFAT2 was attributed to a synergistic interaction of NFAT1 with GATA factors at the IL-13 promoter to stabilize NFAT1 binding (153). In contrast, another report provided evidence for NFAT1 and NFAT2 mediated induction of TNF-α and IL-13 promoters irrespective of which NFAT family member was expressed (154).

IL-9 is a pleiotropic cytokine implicated in mast cell development and an important mediator of allergic diseases (125, 155, 156). It was first characterized as a T-cell and mast cell growth factor and was termed as P40 due to its molecular weight (157). It was previously observed that IL-9 along with activated c-kit and FcεRI enhances expression of proteases and other inflammatory cytokines in mast cells (158).

Involvement of IL-9 in mast cell mediated diseases has spurred interest in various groups to explore its transcriptional regulation in allergic mouse models where it is expressed in several cell types including mast cells. A recent study from our group has analyzed IL-9 gene regulatory element (IL-9 CNS-25) in mast cells and basophils. The CNS-25 enhancer was found to be a potent regulator of transcriptional and epigenetic modification at the IL-9 gene locus (159). The study further elaborated preferential binding of STAT5 and GATA1 to the CNS-25 enhancer compared to the IL-9 promoter in mast cells and a requirement for GATA1 in IL-9 production (Figure 2). Thus, both STAT5 and GATA1 contribute to IL-9 production in mast cells.

A review on IL-9 regulation from our group elaborates the various transcription factors that activate IL-9 gene in mast cells and T cells (160). LPS and IL-1 stimulation markedly induced IL-9 production from mast cells. Notably, the IL-9 promoter harbored binding sites for key transcription factors such as NF-κB and GATA1 via p38 MAP kinase dependent pathway (161–163). The transcription factors regulating mast cell cytokine genes are only beginning to be explored and require more in depth understanding of mechanistic pathways that contribute to their functional roles. Therefore, further advances in defining the role and targets of various transcription factors will promote clarity in the regulation of key genes associated to mast cell functions.

## Concluding Remarks

Transcription factors play a critical role in mast cell development, survival, and function during physiological and pathological conditions. Considerable progress has been made to understand the activity and impact of these factors on mast cell-dependent allergic functions. Table 1 summarizes the key transcription factors and the impact of their deficiency on mast cell phenotypes. Several of these factors work in a cooperative manner along with other transcription factors and chromatin modifying proteins to control their target gene expression. Table 2 summarizes the structural features and biochemical functions of key transcription factors that regulate maturation and function of mast cells. How chromatin in mast cells differs among various tissue sites and how that compares to other cells types has still not been extensively examined. This might yield further insights into the specialization of tissue-specific mast cell functions. Additional efforts to define these protein partners will facilitate identification of novel targets and clinical approaches for mast cell pathologies. Further areas of investigation to study the mechanism regulating the network of these transcription factors in mast cells will lead to better understanding of the pro-allergic functions of mast cells.

**Table 2:** Structural features and biochemical functions of transcription factors.

**Transcription factor**	**Structural features**	**Consensus DNA binding sequence**	**Biochemical functions**	**Reference**
GATA family	Zinc finger DNA binding proteins	(T/A)GATA(A/G)	proliferation and maintenance of hematopoietic and mast cells	(47)
Ets family	winged-helix-turn-helix motif containing protein	purine rich “GGA” core trinucleotide	regulate cell growth, apoptosis, development, differentiation and oncogenic transformation	(63, 64)
MITF	helix-loop-helix (HLH)domain containing protein	M-boxes (5′-TCATGTGCT-3′)	differentiation of common basophil/mast cell committed progenitors (BMCPs) into mast cells	(164)
BATF	basic leucine zipper transcription factor	TGA(C/G)TCA) or (CRE: TGACGTCA)	differentiation of lymphocyte lineage cells (B cells, Th cells and mast cells)	(165)
STAT5	member of JAK-STAT pathway	TTCN3GAA on Bcl-x promoter	cell differentiation, lymphocyte and mast cell development	(166–168)
AP-1	basic leucine zipper (bZIP) proteins	TGAG/CTCA	differentiation, proliferation, and apoptosis	(112)

## Author Contributions

MS wrote the manuscript and MK edited the manuscript.

## Conflict of Interest

The authors declare that the research was conducted in the absence of any commercial or financial relationships that could be construed as a potential conflict of interest.

## Abbreviations

CBMC: Cord Blood–derived Mast Cells
CCR1: Chemokine Receptor type 1
CREB3l1: CAMP Responsive Element Binding Protein 3 Like 1
CRTC3: CREB Regulated Transcription Coactivator 3
CXCR1: C-X-C Chemokine Receptor type 1
EGR2: Early Growth Response 2
Ehf: Ets Homologous Factor
mMC-CPA: Mouse Mast Cell- Carboxypeptidase A
NFATC2, Nuclear Factor of Activated T-cells: Cytoplasmic 2
PIAS3: Protein Inhibitor of Activated STAT3
PKCI: Protein Kinase C Interacting
RUNX1: Runt-related Transcription Factor 1
TOX2: TOX High Mobility Group Box Family Member 2.
